# An update on the management of chronic hepatitis D

**DOI:** 10.1093/gastro/goz052

**Published:** 2019-10-19

**Authors:** Pir Ahmad Shah, Saad Choudhry, Karen J Campoverde Reyes, Daryl T Y Lau

**Affiliations:** 1 Division of Gastroenterology, Beth Israel Deaconess Medical Center, Harvard Medical School, Boston, MA, USA; 2 Neuroendocrine Unit, Massachusetts General Hospital, Harvard Medical School, Boston, MA, USA

**Keywords:** chronic hepatitis D, HDV therapy, HDV diagnostics, interferon, myrcludex B, lonafarnib

## Abstract

Hepatitis D virus (HDV) infection is associated with severe liver-related morbidity and mortality. The prevalence of HDV is rising especially among people who abuse drugs and immigrants from endemic areas. Reliable diagnostic assays with enhanced sensitivity and specificity are essential for screening at-risk populations. Until recently, interferon has been the only treatment for hepatitis D. Its efficacy is, however, limited and it is associated with significant side effects. A number of novel antiviral agents that target various stages of the HDV life cycle show promising results. They are currently in different phases of clinical development. This review focuses on the changing epidemiology, novel therapeutic agents, and updated management of chronic hepatitis delta.

## Introduction

Hepatitis D virus (HDV) has a rod-like genome consisting of approximately 1,700 nucleotides and is the smallest single-stranded RNA virus that can infect humans. HDV requires hepatitis B surface antigen (HBsAg) to replicate [1]. Both hepatitis B virus (HBV) and HDV enter the hepatocytes via the binding of HBsAg to the sodium taurocholate co-transporting polypeptide (NTCP) receptor [2]. HDV shares common modes of transmission as HBV and can be acquired either as coinfection or superinfection to HBV [3, 4]. Dual HBV-HDV coinfection is usually associated with severe and progressive liver disease [5]. It was once thought that HDV was on the verge of eradication but, as migration patterns and high-risk behaviors change worldwide, its epidemiology has also evolved. Accurate and efficient methods for the screening, diagnosis, and management of HDV are essential. This review will focus on the epidemiology, diagnosis, and management of HDV.

## Epidemiology and demographics 

Eight different HDV genotypes have been identified in various geographic regions. HDV genotype 1 has global distribution; genotypes 2 and 4 usually present in the Far East, while genotype 3 is prevalent in northern South America and genotypes 5–8 in Africa [6]. Prior to the 1990s, it was estimated that approximately 15 million (5%) HBsAg carriers worldwide were infected with HDV [3]. By the mid-1990s, HDV prevalence had decreased significantly due to HBV vaccination and AIDS-awareness programs in the Western countries. In the past two decades, however, HDV prevalence has remained high among immigrants from HDV-endemic areas such as Africa and Middle Eastern countries and among people who inject drugs (PWID). It was reported that >70% of the HDV cases in Greece, Hannover, and London were from the immigrant populations [3]. Similarly, PWID accounted for >70% of the HDV infection in the UK, Spain, the Czech Republic, and Germany [3]. The increased detection of HDV cases was also noted among PWIDs in North America. In a study conducted in San Francisco, about 35% of HBsAg-positive injection drug users were identified to have HDV coinfection [7]. Another study from Baltimore, USA, reported an increase in prevalence of HDV from 29% to 50% among the PWIDs between 1989 and 2006 [8]. In fact, a recent systematic review estimated an alarmingly high HDV prevalence of about 62–72 million people globally [9].

## Diagnostic tools for HDV 

In the clinical setting, enzyme-linked immunosorbent assay (ELISA) for anti-HDV is the first-line screening test for hepatitis D. According to the published studies, >90% of patients become anti-HDV-positive within the first 2 months of acute HDV infection [10]. During the acute phase of HDV infection, IgM anti-HDV is transiently detectable in serum. The presence of IgG anti-HDV antibodies indicates chronic HDV infection or serves as a serological marker of past infection with recovery. Screening for HDV antibodies is indicated for HBsAg-positive patients, especially among the high-risk populations such as PWID, men who have sex with men, and immigrants from HDV-endemic regions. The existing commercial ELISA HDV-screening assays have limited availability in developing regions and the test accuracy can be influenced by HDV genotypes [11–14]. The performance of the HDV ELISA assays could contribute to the differences in HDV prevalence and incidence rates in various geographical regions. For example, a study conducted in Amman reported a very high rate of anti-HDV IgM in 83% of patients with chronic hepatitis B [15]. Such a high incidence was not noted in other reports. Recently, a novel quantitative microarray antibody-capture assay has been developed [16]. It has improved the sensitivity and specificity compared to current ELISA assays across the different HDV genotypes.

Individuals who test as anti-HDV-positive should be further confirmed to have active infection with replicative virus by the detection of HDV RNA [11, 14]. Nucleic-acid amplification techniques (NATs) to detect HDV RNA are the most sensitive to confirm active disease and determine treatment response [12, 17]. Serum HDV RNA can be detected by both qualitative and quantitative reverse transcription-polymerase chain reaction (RT-PCR) assays. The nucleotide sequences differ by about 22%–38% between different HDV genotypes [18]. There is, therefore, heterogeneity in the sensitivity and specificity of RT-PCR assays across the HDV genotypes. Standardization of the assays is necessary so the results can be compared and validated from different laboratories [13, 14, 16, 19]. This is particularly problematic in comparing the response rates of earlier therapeutic clinical trials. In 2013, the World Health Organization (WHO) developed an international standard for HDV RNA quantification [12, 13]. The availability of HDV RNA quantification assays is, however, limited, especially in the developing countries where HDV is endemic.

HDV requires HBsAg for complete replication and transmission. HBsAg seroclearance signifies resolution of HDV infection. HBV encodes three envelope proteins: the small (SHBsAg), middle (MHBsAg), and large (LHBsAg) [20]. The quantitative HBsAg (qHBsAg) titers were found to correlate with HDV RNA levels in chronic HDV carriers [21]. Architect QT assay (Abbott Laboratories), the Elecsys HBsAg II Quant assay (Roche Diagnostic), and DiaSorin Liaison XL are some commercialized qHBsAg assays. The current qHBsAg assays are unable to differentiate between the three HBsAg subtypes or integration-derived HBsAg proteins [20].

HDV infection is highly pathogenic, with aggressive progression to cirrhosis. Patients with HDV-induced cirrhosis are at increased risk of hepatocellular carcinoma (HCC) and liver-related mortality compared to HBV mono-infected patients [22]. Routine HDV antigen immunostaining of liver tissue is unavailable and the diagnosis can be missed if relying on only histological features. This underscores the importance of HDV screening among patients with chronic hepatitis B, especially among hepatitis B e-antigen (HBeAg)-negative patients with low HBV DNA levels and elevated serum aminotransferases or hepatic synthetic dysfunction.

## Treatment of HDV

HDV is a unique RNA virus that replicates through a double-rolling circling model with host and not via HDV polymerases [23]. Direct inhibition of viral replication with polymerase inhibitors, therefore, is not possible. HDV requires HBsAg for its propagation. Therapeutic agents that reduce HBsAg titer, theoretically, would also be effective in controlling HDV. Until recently, interferon (IFN)-based therapy has been the only treatment with proven efficacy against chronic hepatitis D. There is a dire need for HDV-treatment modalities, since IFN is associated with potential significant side effects. Furthermore, it is contraindicated for patients with decompensated cirrhosis, active psychiatric conditions, and autoimmune diseases. With the recent advances in the understanding of the molecular replication mechanisms of HBV and HDV, there are some novel and promising antiviral agents in the early phases of development.

### Interferon-based therapy

#### Type I interferons

There is accumulating evidence that HDV is an immune-mediated disease. The earliest studies applying IFNs in HDV patients were conducted in the late 1980s. The interferon-based therapy consists of the use of standard or pegylated IFN alpha (PegIFN-α). The ideal treatment endpoint is the eradication of both HDV and its helper HBV. This remains a major challenge. Six months after the end of therapy, undetectable HDV RNA is the most commonly used surrogate marker of treatment efficacy. This treatment endpoint, however, may not represent a sustained virologic response, as delayed viral clearance or relapse after IFN therapy can occur [18, 24, 25]. With standard IFN-α, high-dose (5 MU daily or 9 MU thrice weekly) IFN for 12 months was associated with 50% sustained biochemical response and longer disease-free survival compared to low-dose regimens [26, 27]. With the long-acting once-weekly PegIFN, small study series from Europe reported a post-treatment virologic response from 17% to 43%. In the HIDIT-1 trial, 90 patients from Germany, Turkey, and Greece were randomized to receive PegIFN-α-2a 180 µg weekly with and without adefovir for 48 weeks [24]. The 6-month post-treatment virologic response rate was 28% with PegIFN-α-2a monotherapy. The addition of adefovir did not improve the treatment outcome. Similarly, there was no increase in virologic response when nucleoside analogs such as lamivudine or ribavirin were used in combination with standard or PegIFN-α [18, 28, 29]. Nucleoside/nucleotide analogs are HBV polymerase inhibitors that cause primarily an inhibition of HBV DNA synthesis. They do not directly suppress the production of HBsAg, which is the primary helper function of HBV in the HDV life cycle.

Based on the published clinical trial results on PegIFN, chronic hepatitis D should be treated for at least 1 year. The optimal treatment duration, however, has not been well established. A number of studies includes the HIDIT-2 treatment trial, in which prolonged administration of PegIFN for 2 years did not improve the post-treatment virologic response [30]. There are limited but convincing reports that long-term IFN-based therapy is associated with regression of hepatic fibrosis, HDV RNA, and HBsAg clearance [31, 32]. Since interferons can be associated with significant adverse events, consideration between prolonged therapy and drug toxicity is critical. It is essential to monitor patients carefully for side effects such as flu-like symptoms, infection, depression, neutropenia, thrombocytopenia, and thyroid dysfunction.

Studies have shown that viral kinetics, quantitative HBsAg, and RNA have a role in predicting HDV virological response. One study, for example, found that HBsAg <1,000 IU/mL at month 6 of interferon-based therapy differentiated responders and partial responders from non-responders (*P *<* *0.001) [33]. However, in another study, HBsAg titers at week 24 of therapy were significant only in univariate analysis [34] .This could be secondary to the different quantitative assays used in the studies. Standardized serological and molecular assays are needed to compare treatment responses from various trials.

Kinetic of HDV RNA has also been used to predict treatment response. A study reported that a 2 log reduction in HDV RNA levels at week 24 of therapy had a sensitivity of 92% and a specificity of 74% for predicting virological response. Furthermore, a <2 log decrease in HDV RNA on treatment had a 95% negative predictive value for null response in another study [34].

#### Type III interferon: interferon-lambda

Peg IFN-α is associated with significant side effects as type I interferon receptors are distributed throughout the body. Because HDV infection is restricted to the liver, interferon-lambda (IFN-λ) represents an attractive alternative, since the type III interferon receptors are selectively expressed on hepatocytes in high concentration [35]. A phase 2 lambda interferon monotherapy (LIMT) HDV study is a randomized, open-label, multicenter trial. A total of 33 patients with chronic hepatitis D were randomized to receive IFN-λ 180 µg (*n *=* *14) or 120 µg (*n *=* *19) for 48 weeks with 24 weeks’ follow-up. All patients received nucleoside/nucleotide analogs for HBV during the entire treatment period. At week 48, 7 of 14 (50%) patients treated with 180 μg lambda experienced a ≥2 log decline and 36% achieved HDV RNA negativity at the end of treatment [36]. The results were comparable to historical PegIFN for HDV-infected patients. Mild to moderate flu-like symptoms and elevated transaminase levels were reported but there were few episodes of cytopenia [36]. Cases of hyperbilirubinemia were noted in the Pakistani cohort without hepatic decompensation. All patients responded to medication dose reduction or discontinuation. Further studies are being conducted to further evaluate the efficacy and safety of IFN-λ in combination with other therapeutic agents.

### Emerging new therapy

#### Myrcludex B (hepatocyte entry inhibitor)

HBV and HDV enter the hepatocytes through the NTCP receptors [2]. Myrcludex B (Myr B) is a myristoylated lipopeptide comprising 47 amino acids of the pre-S1-domain of the HBV L-surface protein. In earlier clinical trials, Myr B demonstrated activity against both hepatitis B and D, leading to a significant decline in the viral load of both viruses [37]. In a multicenter, open-label phase 2 trial (MYR202), 120 patients were treated with either tenofovir alone or Myr B (at 2, 5, or 10 mg) in combination with tenofovir for 24 weeks to assess its safety and efficacy. At the end of therapy, there was a dose-dependent decline in HDV RNA between 1.6 log to 2.7 log in the Myr B-treated arm. There was, however, no reduction in HBsAg levels. At 12 weeks of follow-up, 80% of the patients who responded to Myr B had relapse of HDV RNA [38]. Another phase 2 trial was designed to evaluate the efficacy of Myr B and pegIFN-α-2a combination therapy. Sixty HBeAg-negative patients with HBV/HDV coinfection were divided into four groups: 180 µg pegIFN-α alone, Myr B alone, Myr B 2 mg with PegIFN-α, or Myr B 5 mg with PegIFN-α for 48 weeks. Combination therapy with both doses showed strong synergism with a median reduction in HDV RNA of 3.62 log in the 2 mg Myr B group and 4.48 log in the 5 mg group at 48 weeks. In contrast, the PegIFN-α and Myr B monotherapy groups only achieved a median HDV RNA reduction of 1.14 log and 2.84 log, respectively. The combination therapy was associated with HBsAg-level decline of >1 log or undetectable in 9/30 of patients [38]. No serious adverse event was reported. An increase in the bile-acid level, however, was reported in all the studies with Myr B. More evaluation of the safety profile of Myr B is required, as bile acids have been implicated in cardiac arrhythmias [37].

#### Lonafarnib (prenylation inhibitor)

Prenylation is the process by which prenyl farnesyl transferase attaches a lipid group to the HDV large antigen (HDLAg) [39]. That is a critical step for interaction of HDLAg with HBsAg and formation of the secreted viral particles. Lonafarnib (LNF) is a farnesyl transferase inhibitor that was noted to be effective against HDV in both *in vivo* and *in vitro* studies. In an early proof-of-concept study, a 28-day trial was conducted to evaluate the efficacy of LNF at 100- and 200-mg doses. The mean decrease in HDV RNA was 0.73 log and 1.54 log for the 100- and 200-mg dose, respectively. It was noted that the drop in HDV RNA was directly correlated with the serum concentration of LNF in the patients. Gastrointestinal symptoms such anorexia, nausea, vomiting, and diarrhea were the major side effects, especially with the 200-mg dose [40].

Ritonavir (RTV) is an inhibitor of the cytochrome p450 enzyme CYP3A4, which metabolizes lonafarnib. RTV, therefore, increases the LNF plasma level by decreasing its metabolism. In previous studies, the side effects of LNF were noted to be dose-dependent. Four phase 2 studies were designed to examine the effect of RTV on LNF bioavailability, efficacy, and side-effect profile at low doses while maintaining its serum concentration. These trials were called ‘Lonafarnib with and without ritonavir HDV studies’ (LOWR HDV).

The LOWR HDV-1 study demonstrated that co-administering LNF with RTV increases the serum level of LNF, achieving greater efficacy with LNF doses compared to LNF monotherapy [41].

The aim of the LOWR HDV-2 study is to identify optimal combination regimens of LNF and RTV with and without PegIFN-α. Patients were assigned to three arms: low dose (25 or 50 mg twice daily [BID]) LNF + RTV 100 mg BID for 24 weeks; high dose (≥75 mg BID) LNF + RTV 100 mg BID for 12 weeks; low dose (25 or 50 mg BID) LNF + RTV 100 mg BID + PegIFN-α 180 µg once-weekly (QW) for 24 weeks. Low-dose LNF regimens were found to have comparable antiviral efficacy with fewer gastroenterological side effects than the higher-dose regimens. The subjects on combination therapy with PegIFN-α achieved the highest rate of HDV RNA suppression and undetectable HDV RNA at the end of therapy [42]. The results confirmed the synergistic efficacy of LNF and PegIFN-α.

LOWR HDV-3 explored single daily doses of LNF (50 vs 75 vs 100 mg) + RTV (100 mg) for up to 24 weeks. The once-daily RTV-boosted LNF was safe and tolerable in patients for up to 6 months of continuous therapy [43]. The LOWR HDV-4 study [44] is an open-label dose-escalation study of LNF + RTV to evaluate whether rapid step-wise increases in LNF from 50 mg BID to 100 mg BID can lead to better tolerability of higher doses. Ten of 15 patients (66%) were able to escalate LNF to 100 mg BID but only 5 were able to maintain the high dose for up to 24 weeks. All patients had HDV RNA decline and one achieved undetectable HDV RNA on therapy. The alanine aminotransferase (ALT) normalized in 53% of patients but five experienced post-treatment hepatitis flare with normal synthetic function. An individualized LNF dose regimen with RTV is a possible strategy to overcome gastrointestinal adverse effects and prolonged therapy duration to achieve optimal virological response. Post-treatment virological response and hepatitis flare need to be carefully monitored.

#### REP 2139 (nucleic-acid polymer)

The nucleic-acid polymers inhibit the secretion of subviral particles from the hepatocyte. They may also have an effect on the early steps of the HBV replication. They have demonstrated antiviral activities against both HBV and HDV. The exact mechanism of action of this class of drugs, however, is not fully understood. In a phase 2 pilot study, 12 HBV-infected patients were given a weekly intravenous infusion of REP 2139 for 20–35 weeks. In three of those patients, HBsAg levels decreased below the detection limit [45]. With these promising results, another trial on HBV and HDV co-infected patients was conducted. Twelve co-infected patients received weekly intravenous (IV) REP 2139-Ca (500 mg) for 15 weeks followed by 15 weeks of IV REP 2139-Ca (250 mg) combined with PegIFN-α and finally PegIFN-α alone for 33 weeks. In 4 of 12 (33%) patients, their HDV RNA became undetectable by the end of therapy. The decreased HDV RNA level was associated with a 5 log reduction of serum HBsAg titer. After discontinuation of therapy, HDV RNA increased back to baseline levels in 3 of 12 patients [46]. More extensive studies are critical to understanding the mechanisms of action and safety profile of this promising medication. In animal studies, the inhibition of HBsAg secretion was associated with a potential increase in HCC risk [47, 48]. It is unknown whether that is relevant with this class of medication.

## Management strategy for chronic hepatitis D

**Figure 1.**
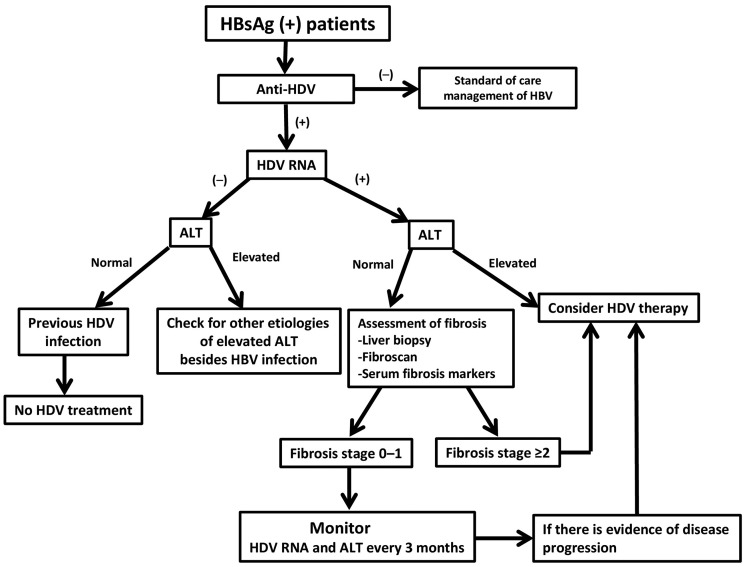
Algorithm for the evaluation of chronic hepatitis D

There is no Food and Drug Administration (FDA)-approved therapy for chronic hepatitis D. The treatment recommendations to date are based on the experience with IFN-based therapy. According to the American Association for the Study of Liver Diseases (AASLD) treatment guideline, HDV screening should be performed among HBsAg-positive individuals who have high-risk behaviors such as PWID, men who have sex with men, human immunodeficiency virus-infected patients, and immigrants from areas with high HDV prevalence [14]. HDV screening should also be considered in patients with hepatitis B whose HBV DNA titers are <2,000 IU/mL but have elevated ALT levels [14]. It is the practice of the authors to routinely screen all HBsAg-positive patients for HDV regardless of risk factors (Figure 1). For those whose anti-HDV is tested negative, they should receive standard-of-care management of chronic hepatitis B according to treatment guidelines [14]. If anti-HDV is screened as positive, quantitative HDV RNA should be measured by RT-PCR to identify patients with replicative virus. Individuals with undetectable HDV RNA and normal ALT level have spontaneous recovery from HDV infection and do not require therapy. Other etiologies of liver diseases, besides HBV infection, need to be considered for those with undetectable HDV RNA but elevated ALT levels.

**Figure 2.**
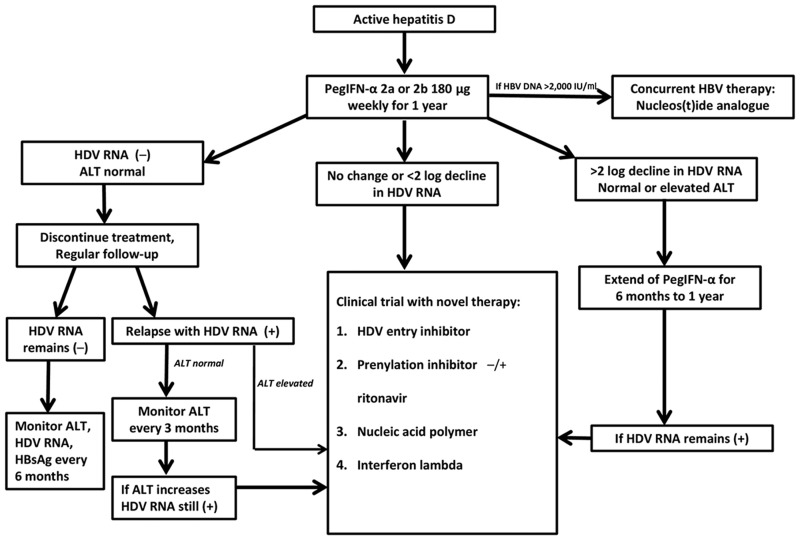
Algorithm for the treatment of chronic hepatitis D

For patients with active hepatitis D—namely HDV RNA-positive with elevated ALT—HDV therapy should be initiated (Figure 2). Individuals with detectable HDV RNA but normal ALT should have hepatic-fibrosis assessment. Until recently, liver biopsy was the standard modality in assessing liver histology but it is an invasive procedure. Fibroscan and serum markers have been applied in clinical settings to identify patients for therapy and to monitor treatment response. These non-invasive methods do have a role in the management of hepatitis D but need to be more thoroughly validated [49]. Generally, treatment is indicated for those with moderate fibrosis stage ≥2. Patients with detectable HDV RNA and hepatic-fibrosis stage 0–1 may be monitored every 3–6 months with ALT and liver-function tests. If there is evidence of disease progression, HDV therapy needs to be promptly initiated (Figure 2).

In our opinion, patients with active hepatitis D with HDV RNA positive and elevated ALT or hepatic fibrosis ≥2, treatment with PegIFN-α-2a or PegIFN-α-2b 180 µg weekly should be initiated if there is no contraindication. Concurrent nucleoside/nucleotide analogs for HBV is strongly recommended especially if the HBV DNA level is >2,000 IU/mL to prevent HBV reactivation after HDV treatment.

After 1 year of therapy, if HDV RNA becomes undetectable with normal ALT, IFN treatment can be discontinued and responders should be monitored with HDV RNA and ALT every 3–6 months for 1 year and every 6 months thereafter. During monitoring, if hepatitis D relapses with positive HDV RNA and elevated ALT, experimental therapies for HDV should be considered. However, if ALT remains normal despite detectable HDV RNA, close monitoring of ALT every 3 months is indicated. Experimental therapies are reasonable options if ALT becomes elevated during follow-up.

Experimental therapies should also be considered for non-responders with <2 log decline in HDV RNA after 24 weeks. If a patient achieves >2 log decrease but detectable HDV RNA at the end of 1 year of therapy, PegIFN-α can be extended for another 6 months to 1 year. If HDV RNA is still positive after extended PegIFN-α therapy, clinical trial with novel therapy is strongly recommended.

## Conclusion

Hepatitis D is a major public-health problem globally. In developed countries, vaccination and health-awareness programs have largely contained the epidemic. Hepatitis D remains a significant illness for PWID as well as immigrants from endemic areas. Prevalence of HDV needs to be more extensively studied so effective disease-awareness programs can be implemented by focusing on at-risk populations. Reliable assays with high sensitivity and specificity need to be validated and commercialized for timely diagnosis and management of hepatitis D. There are a number of promising novel therapies in development that target various stages of HDV replication. Hopefully, HDV eradication will become a reality with both preventive measures and improved therapy.

## Authors’ contribution

P.A.S. and D.T.Y.L. contributed to the concept and design of the review, the writing and final approval of the manuscript. S.C. focused on researching on the new therapy section. K.J.C.R. focused on the epidemiology and diagnosis aspects of the study.

## Funding

None.
